# Rhythmic light exposure during incubation enhances liver development and lipid accumulation in chicken embryos

**DOI:** 10.5713/ab.25.0248

**Published:** 2025-07-11

**Authors:** Xiuyu Xie, Fang Li, Jiayi Zhang, Dan Song, Yongshu Wu, Lu Liu, Yanjun Cui, Xiangchen Li, Panlin Wang

**Affiliations:** 1 College of Animal Science and Technology & College of Veterinary Medicine, Zhejiang A&F University, Key Laboratory of Applied Technology on Green-Eco-Healthy Animal Husbandry of Zhejiang Province, Hangzhou, China

**Keywords:** Chicken, Circadian Rhythm, Light Environment, Lipid Metabolism, Liver

## Abstract

**Objective:**

Chicken embryos are typically incubated in complete darkness, overlooking the potential influence of rhythmic light exposure. This study aimed to investigate the effects of a 12-hour light/12-hour dark rhythmic light environment on liver development and lipid metabolism in chicken embryos, providing a foundation for regulating poultry physiology through light manipulation.

**Methods:**

Fertile eggs were incubated under two lighting conditions: a 12 h light/12 h dark cycle (LD group) and constant darkness (DD group). Embryos or chicks were sampled at embryonic days 12 (E12), 14 (E14), 16 (E16), 18 (E18), 20 (E20), and day 1 post-hatch (D1). Body weight, liver weight, and residual yolk weight were recorded. Liver morphology and lipid accumulation were evaluated using H&E and Oil Red O staining. Hepatic levels of triglycerides (TG), total cholesterol (TC), free fatty acids, fatty acid synthase (FAS), and acetyl-CoA carboxylase (ACC) were measured. Expression of lipid metabolism-related genes was assessed by real-time quantitative polymerase chain reaction.

**Results:**

Embryo weight, liver weight, liver index, and residual yolk weight did not differ significantly between groups. However, liver tissues in the LD group exhibited earlier maturation of liver plate structures and increased lipid droplet accumulation. TG concentration was significantly higher at E12 and D1, TC at E12 and E18, and ACC at E12, E16, and E18 in the LD group (p<0.05). Furthermore, lipid synthesis genes (*ACC*, *FAS*, *SCD1*, *SREBP-1c*, *ELOVL6*) were upregulated, while lipid degradation genes (*CPT1*, *PPARα*, *MTTP*) were downregulated at specific embryonic stages in the LD group (p<0.05).

**Conclusion:**

Rhythmic light exposure under a 12 h light/12 h dark cycle promoted liver development and lipid accumulation by modulating expression of lipid metabolism genes such as ACC, FAS, and CPT1. These findings highlight the potential of light rhythm as a strategy to optimize embryonic development and lipid metabolism in poultry.

## INTRODUCTION

Studies show that some chicks may go without feeding for up to 72 hours after hatching due to variations in hatching times, along with factors like immunization and transportation [1,2]. During this period, their energy primarily comes from hepatic lipid breakdown. Therefore, liver lipid reserves during embryonic development and hatching play a key role in their ability to cope with external challenges. The circadian clock’s regulation of lipid metabolism is well-established in mammals [3,4]. However, poultry embryos, typically incubated in darkness, lack access to circadian rhythm signals, and research on how incubation light affects hepatic lipid metabolism remains limited.

In natural incubation environments, hens forage during the day, providing periodic light exposure to the embryos. Studies have shown that rhythmic light exposure during incubation reduces long-term fear in chicks and promotes leg health [5]. Our previous research found that 12-hour light/12-hour dark (12L:12D) rhythmic light exposure helps embryos establish a circadian clock in the pineal gland, promoting melatonin secretion [6,7]. It also enhances insulin-like growth factor 1 (IGF-1) secretion in the liver, accelerates embryonic development, and advances hatching time [7,8]. Additionally, we observed that chicken embryos incubated under 12L:12D rhythmic light environment exhibited circadian rhythms in liver lipid metabolism, with increased triglycerides (TG), free fatty acids (FFA), total cholesterol (TC), and fatty acid synthase (FAS) during the light period, and enhanced breakdown during the dark period—this phenomenon was not observed in total darkness [9]. These findings suggest that rhythmic light exposure during incubation may play a significant role in regulating hepatic lipid metabolism. However, our previous study mainly focused on the rhythmic patterns of hepatic lipid metabolism over a 24-hour cycle under different light conditions, without evaluating the actual lipid reserves in the liver at different embryonic stages or in newly hatched chicks. Therefore, the present study aimed to determine how rhythmic light exposure affects the dynamic accumulation of hepatic lipid reserves throughout embryonic development and at hatch.

Liver lipid metabolism is co-regulated by multiple genes. In the fatty acid synthesis pathway, acetyl-CoA carboxylase (ACC), as a rate-limiting enzyme, catalyzes the conversion of acetyl-CoA to malonyl-CoA [10]. FAS is a key enzyme in fatty acid synthesis, mediating the formation of long-chain fatty acids through the condensation of acetyl-CoA and malonyl-CoA [10]. ELOVL6 and stearoyl-CoA desaturase 1 (SCD1) are involved in the elongation and desaturation of fatty acid chains [11,12]. The expression of these genes is activated by transcription factors, including carbohydrate-responsive element-binding protein (ChREBP) and sterol regulatory element-binding protein 1c (SREBP-1c) [13]. In terms of catabolism, carnitine palmitoyl transferase-1beta (CPT1) mediates the initial steps of fatty acid β-oxidation [14], while peroxisome proliferator-activated receptor α (PPARα) regulates the expression of fatty acid oxidation gene clusters [15]. The microsomal triglyceride transfer protein (MTTP) facilitates the transport of TG [16]. Previous studies have indicated that light cycles can influence the expression of SREBP-1c in quail liver and the accumulation of TG [17]. The core circadian clock proteins circadian locomotor output cycles kaput (CLOCK) and brain and muscle ARNT-like 1 (BMAL1) form heterodimers that can directly bind to the E-box element in the PPARα promoter, thereby enhancing its transcription [9]. In mice, knockout of the liver clock genes BMAL1/CLOCK weakened the ability of piperine to alleviate the overexpression of lipid metabolism-related genes, such as SREBP-1c, FAS, and ACC, induced by oleic acid [18]. In continuous darkness, PPARγ expression is upregulated in zebrafish larvae [19]; deletion of the CLOCK gene in mouse liver can downregulate SREBP expression [20]. These findings suggest that light cycles may regulate lipid metabolism gene expression through circadian clocks, thus influencing fat accumulation. However, the regulatory effects of the 12L:12D rhythmic light environments on hepatic lipid metabolism-related gene expression in chicken embryos are still unknown.

This study aims to compare the effects of constant darkness and 12L:12D cycles on liver development dynamics and lipid metabolism in chicken embryos and newly hatched chicks, while also elucidating the regulatory influence of light environments on key lipid metabolism genes.

## MATERIALS AND METHODS

### Experimental design

Fertilized Sanhuang chicken eggs were used as experimental materials, with an average egg weight of 43±2.0 g. All eggs were collected within five days and stored at 15±1°C and 70%–75% humidity for no more than seven days before incubation. To eliminate light interference, blackout curtains were used to cover both the front windows of the incubator and the windows of the incubation room. A total of 360 eggs were randomly and evenly assigned to two experimental groups. The DD group was incubated under complete darkness, while the LD group was exposed to a 12L:12D photoperiod using white LED strips (450 lux) fixed on the racks of each floor, ensuring uniform light exposure to all eggs. Except for the light conditions, all other incubation parameters were identical between two groups. During the first 18 days of incubation, the eggs were turned every hour, with the incubator maintained at 37.8±0.1°C and ~60% humidity. On day 19, egg turning was ceased, and the eggs were transferred to hatching trays. The incubator temperature was then adjusted to 37.2±0.01°C, with humidity increased to 70%, where it remained until hatching was complete.

### Sample collection

Embryos, livers, and residual yolks were collected from both groups at embryonic days (E) 12, 14, 16, 18, 20, and 1 day post-hatch (D1). At least six eggs per group were sampled at each time point. After removing surface moisture with filter paper, the weights of the embryo, residual yolk, and liver were recorded to evaluate growth and development. For histological analysis, approximately 1 cm^3^ of liver tissue from E16, 18, 20, and D1 embryos was fixed in 4% paraformaldehyde for subsequent hematoxylineosin (HE) staining and Oil Red O staining. Liver tissues from all sampled embryonic stages were flash-frozen in liquid nitrogen and stored at −80°C for biochemical analysis of lipid metabolism and gene expression profiling.

### Embryonic growth and development

The body weight, liver weight, and residual yolk weight of embryos at E12, 14, 16, 18, 20, and D1 were measured to evaluate embryonic growth patterns. The liver index was determined using the following formula: Liver index = Liver weight (g)/Embryo weight (g) [21]. To ensure accurate measurement of liver weight, liver tissues were carefully dissected using fine-tipped forceps, briefly rinsed in phosphate-buffered saline to remove residual blood, gently blotted dry with absorbent paper, and immediately weighed using an analytical balance with a precision of 0.1 mg.

### Hematoxylineosin and Oil Red O staining

Liver tissues fixed in 4% paraformaldehyde were dehydrated, cleared, embedded in paraffin, and sectioned. The sections were deparaffinized and subjected to HE staining. For Oil Red O staining, another set of liver samples was embedded in OCT compound, sectioned into 5–7 μm slices using a Thermo Fisher cryostat and a Leica microtome, and stained according to the manufacturer’s protocol using filtered Oil Red O solution.

### Biochemical analysis of lipid metabolism

Liver tissues from LD and DD groups were thawed, washed with pre-chilled saline, and homogenized using a high-throughput tissue homogenizer. The homogenate was centrifuged at 3,000 rpm for 15 min at 4°C, and the supernatant was collected for biochemical analysis. According to the manufacturer’s instructions (Nanjing Jiancheng Bioengineering Institute), enzyme-linked assays were used to quantify the concentrations of TG, FFA, TC, glucose (GLU), FAS, and ACC in the liver at E12, 14, 16, 18, 20, and D1. Additionally, total protein concentration was determined using a BCA protein assay kit for normalization.

### Gene expression analysis of lipid metabolism

Total RNA was extracted from liver samples using the SteadyPure RNA Extraction Kit (Accurate Biotechnology) and chloroform-based phase separation. Reverse transcription was performed using the EvoM-MLVRT Kit (Accurate Biotechnology) to synthesize cDNA. The expression levels of lipid metabolism-related genes were analyzed using real-time quantitative polymerase chain reaction with the SYBR Green Pro Taq HS Premix (Accurate Biotechnology). Primers were designed as previously described [21], and their sequences are listed in Table 1.

### Statistical analysis

Differences in embryonic growth, lipid metabolism-related biochemical parameters, and gene expression between two groups were analyzed using unpaired t-tests, performed with GraphPad Prism 10.1.2 (GraphPad Software). Each individual embryo at each time point was considered as one experimental unit. Statistical significance was set at p<0.05.

## RESULTS

### Embryonic growth and development

**Figure 1:**
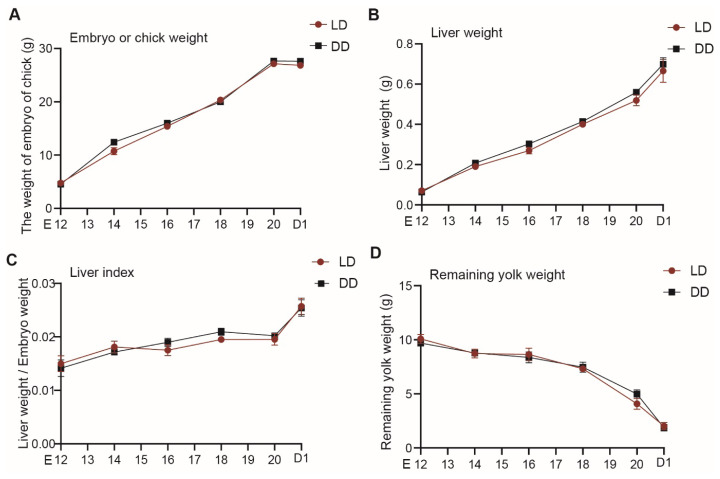
Developmental changes in growth parameters of embryos and chicks. (A) Body weight; (B) Liver weight; (C) Liver index; (D) Residual yolk weight. An asterisk indicates a significant difference between groups at the same developmental stage (* p<0.05). Data are presented as mean±SEM (n = 6 per group). LD, 12L:12D light/dark cycle group; DD, constant darkness group; E12, embryonic days 12; E14, embryonic days 14; E16, embryonic days 16; E18, embryonic days 18; E20, embryonic days 20; D1, day 1 post-hatch; SEM, standard error of the mean.

In both groups, body weight, liver weight, and liver index gradually increased with embryonic age, while residual yolk weight progressively decreased. Between E20 and D1, no significant increase in body weight was observed. However, the liver index showed a more rapid increase, and the depletion of residual yolk accelerated, suggesting a possible acceleration in liver growth during this stage. Additionally, this phenomenon may be associated with water loss and feather drying after hatching. No significant differences were observed between two groups in terms of body weight, liver weight, liver index, or residual yolk weight (p>0.05) (Figure 1).

### Hematoxylineosin and Oil Red O staining

**Figure 2:**
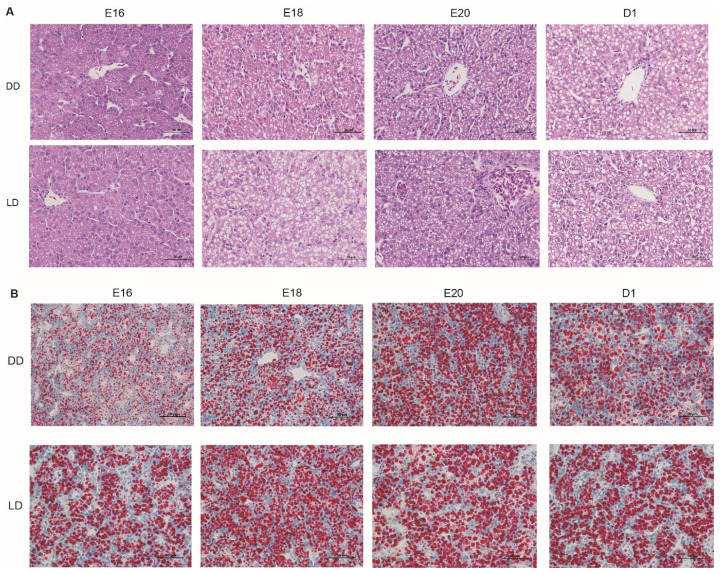
Histological analysis of chick liver from embryonic day 16 to post-hatch day 1 using hematoxylin-eosin (A) and Oil Red O staining (B). E16, embryonic days 16; E18, embryonic days 18; E20, embryonic days 20; D1, day 1 post-hatch; DD, constant darkness group; LD, 12L:12D light/dark cycle group.

**Figure 3:**
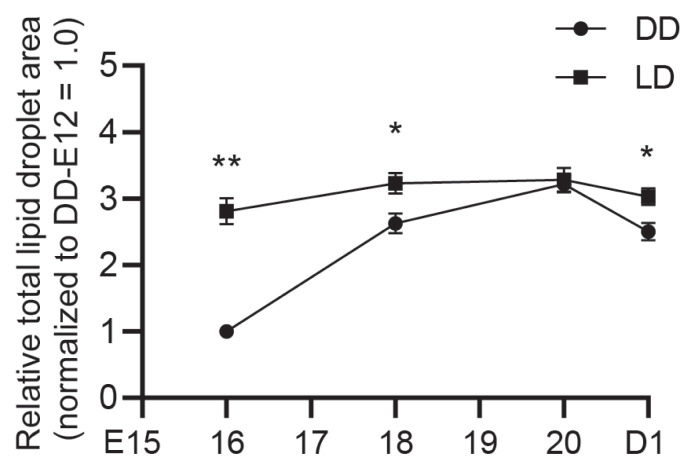
Quantification of hepatic lipid droplet accumulation using Oil Red O staining. Relative total lipid droplet area was normalized to the DD group at E12 (set as 1.0). Asterisks indicates a significant difference between groups at the same developmental stage (* p<0.05; ** p<0.01). Data are presented as mean±SEM (n = 6 per group). DD, constant darkness group; LD, 12L:12D light/dark cycle group; E16, embryonic days 16; E18, embryonic days 18; E20, embryonic days 20; D1, day 1 post-hatch; SEM, standard error of the mean.

In both groups, hepatic plate arrangement became increasingly compact with embryonic age, indicating progressive liver tissue maturation. Compared with the DD group, the LD group exhibited earlier structural maturation of hepatic plates (Figure 2A). Quantitative analysis of Oil Red O staining revealed that lipid droplet deposition in the liver increased with embryonic age, peaked at E20, and slightly declined at D1. Lipid accumulation was significantly greater in the LD group at E16 (p<0.01), E18 (p<0.05), and D1 (p<0.05), as shown in Figures 2B, 3.

### Lipid metabolism-related biochemical indicators

**Figure 4:**
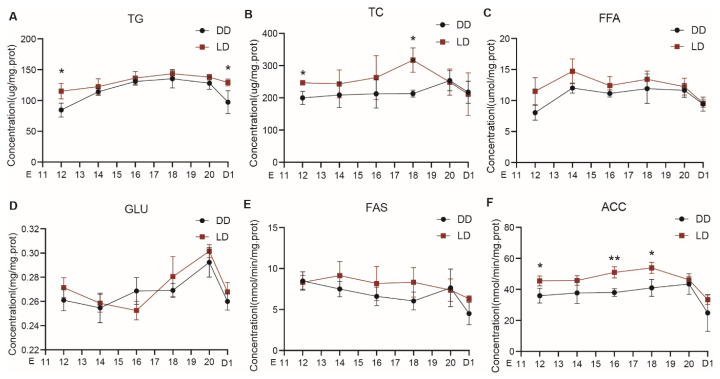
Developmental changes in liver lipid metabolism-related biochemical parameters in embryos or chicks: (A) TG concentration; (B) TC concentration; (C) FFA concentration; (D) GLC concentration; (E) FAS concentration; (F) ACC concentration. An asterisk indicates a significant difference between groups at the same developmental stage (* p<0.05). Data are presented as mean±SEM (n = 6 per group). TG, triglycerides; DD, constant darkness group; LD, 12L:12D light/dark cycle group; TC, total cholesterol; FFA, free fatty acids; GLC, glucose; FAS, fatty acid synthase; ACC, acetyl-CoA carboxylase; SEM, standard error of the mean.

As shown in Figure 4, TG concentrations were significantly higher in the LD group than in the DD group at E12 and D1 (p<0.05), with no significant differences observed at other stages (p>0.05) (Figure 4A). TC concentrations were significantly higher in the LD group at E12 and E18 (p<0.05), while no differences were detected at other stages (p>0.05) (Figure 4B). No significant differences were observed between two groups in FFA, GLU, and FAS concentrations across all stages (p>0.05) (Figures 4C–4E). ACC concentrations were significantly higher in the LD group at E12, E16, and E18 (p<0.05), with no differences at other stages (p>0.05) (Figure 4F). Additionally, all six biochemical indicators exhibited varying degrees of decline from E20 to D1 under both environmental conditions, a trend consistent with the Oil Red O staining results.

### Genes related to lipid metabolism

**Figure 5:**
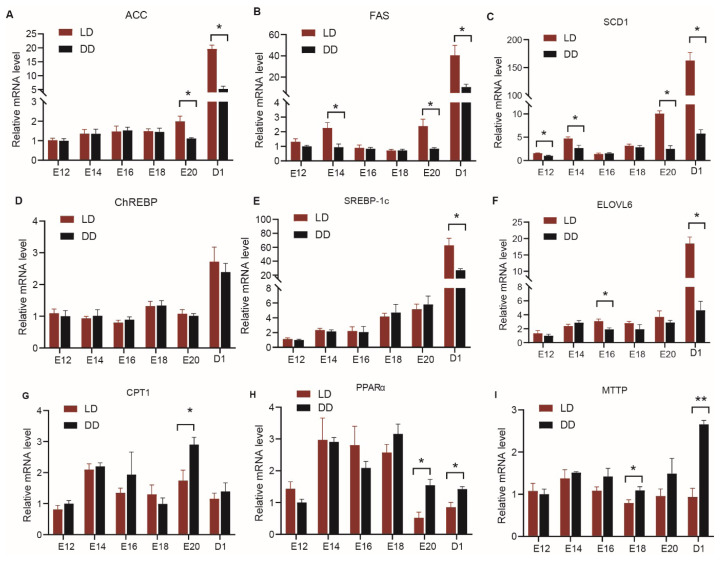
Developmental changes in the expression of hepatic genes related to lipid synthesis and degradation in embryos or chicks. (A) ACC; (B) FAS; (C) SCD1; (D) ChREBP; (E) SREBP-1c; (F) ELOVL6; (G) CPT1; (H) PPARα; (I) MTTP. An asterisk indicates a significant difference between groups at the same developmental stage (* p<0.05). Data are presented as mean±SEM (n = 6 per group). ACC, acetyl CoA carboxylase; LD, 12L:12D light/dark cycle group; DD, constant darkness group; FAS, fatty acid synthase; SCD1, stearoyl-CoA desaturase 1; ChREBP, carbohydrate responsive element binding protein; SREBP-1c, sterol regulatory element binding proteins 1c; ELOVL6, elongase of very long chain fatty acids 6; CPT1, carnitine palmitoyltransferase 1; PPARα, peroxisome proliferator-activated receptor α; MTTP, microsomal triglyceride transfer protein; SEM, standard error of the mean.

As shown in Figure 5, for lipid synthesis-related genes, both groups exhibited low mRNA expression of *ACC*, *FAS*, *SCD1*, *ChREBP*, *SREBP-1c*, and *ELOVL6* in the liver prior to hatching, with a significant increase observed on day 1 post-hatch. Compared to the DD group, the LD group showed significantly higher expression levels of *ACC* at E20 and D1 (p< 0.05), *FAS* at E14, E20, and D1 (p<0.05), *SCD1* at E12, E14, E20, and D1 (p<0.05), *SREBP-1c* at D1, and *ELOVL6* at E16 and D1 (p<0.05) (Figures 5A–5F). For lipid catabolism-related genes, the LD group showed significantly lower expression of *CPT1* at E20 (p<0.05), *PPARα* at E20 and D1 (p<0.05), and *MTTP* at E18 and D1 (p<0.05) compared to the DD group (Figures 5G–5I).

## DISCUSSION

As a photosensitive species, poultry are typically incubated in complete darkness, which neglects the potential circadian requirements for light during critical stages of embryonic development. Elucidating the effects of rhythmic light exposure on liver development and lipid metabolism in chicken embryos is essential for establishing light-based strategies to regulate embryonic metabolism and provides a theoretical basis for improving poultry incubation practices.

The liver plays a central role in energy metabolism during embryonic development, and its developmental status directly influences early post-hatch growth and environmental adaptability in chicks [22]. Previous studies have demonstrated that rhythmic lighting during incubation facilitates the establishment of circadian clocks in poultry [23]. Moreover, exposure to monochromatic light significantly affects melatonin and IGF-1 levels, as well as the expression of hepatic clock genes in Yangzhou geese, ultimately promoting body weight gain [24]. In this study, we observed significant histological dynamics in the developing chicken embryonic liver. As embryonic age increased (E16→D1), the hepatic plates became progressively more compact, indicating gradual liver maturation. Notably, compared to the DD group, the LD group exhibited earlier structural maturation of hepatic plates. This finding aligns with our previous research, which demonstrated that rhythmic green light exposure of 12L:12D during incubation promotes IGF-1 secretion in the embryonic liver and increases liver weight [7]. However, in the present study, we did not observe a significant effect of rhythmic light exposure on liver weight, suggesting that this discrepancy may be related to differences in light wavelength. Previous studies have provided substantial evidence supporting the impact of light environments on organ development. For instance, in zebrafish, continuous darkness, compared to a 14L:10D light cycle, suppresses liver development and reduces gut microbiota diversity [25]. Moreover, the addition of dim light at night under a 12L:12D cycle negatively affects intestinal and hepatic development in male zebra finches [26]. These findings provide strong theoretical support for understanding the regulatory effects of light environments on liver development.

The liver is the primary organ responsible for de novo lipid synthesis in poultry, regulating approximately 95% of total body fat production [27]. During embryonic development and the early post-hatch period, hepatic lipid reserves play a crucial role in supporting growth and development. In the final days before hatching and the first three days post-hatch, chicks primarily rely on hepatic lipid breakdown for energy to sustain basic physiological functions and early growth [28]. However, due to factors such as the hatching window, chick handling, and transportation, newly hatched chicks may experience up to 72 hours without access to feed and water [2,29,30]. Studies have shown that 48 hours of post-hatch feed and water deprivation significantly reduces body weight in layer chicks, accompanied by a marked decline in serum GLU, total protein, and TG levels, with these effects persisting until 56 days of age [31]. Therefore, hepatic lipid reserves during the embryonic and early post-hatch stages are essential for chicks to cope with environmental challenges, maintain vital functions, and support subsequent growth and development. To enhance liver lipid reserves in chicks, previous studies have focused on maternal dietary interventions, such as supplementing conjugated linoleic acid to regulate embryonic hepatic lipid metabolism [32,33]. However, limited research has explored the influence of incubation conditions on this process. Our study found that rhythmic light exposure during incubation significantly enhanced hepatic lipid reserves in chicken embryos. Oil Red O staining showed that lipid droplet accumulation in the embryonic liver was markedly higher under a 12L:12D light-dark cycle compared to constant darkness. Additionally, at specific embryonic stages, the levels of TG, TC, and ACC were significantly higher in the LD group than in the DD group, consistent with the Oil Red O staining results. These findings suggest that rhythmic light exposure during incubation promotes lipid storage in late-stage embryos and newly hatched chicks, offering new insights into optimizing hepatic energy reserves in chicken embryos and hatchlings. Previous research has demonstrated that continuous light exposure disrupts circadian rhythms in zebra finches, abolishing the rhythmic expression of the hepatic *BMAL1* gene and leading to increased fat deposition [34]. Our previous studies further showed that in chicken embryos incubated under a 12L:12D light cycle, hepatic lipid metabolism exhibited a distinct circadian rhythm, with TG, FFA, TC, and FAS levels increasing during the light phase and decreasing during the dark phase, a pattern absent in embryos incubated in complete darkness. These findings suggest that the metabolic effects of rhythmic light exposure may arise from its ability to entrain the embryonic circadian clock. Light acts as a primary environmental cue, aligning circadian gene expression through core components such as CLOCK and BMAL1, which in turn regulate downstream genes involved in lipid synthesis and degradation. This circadian regulation likely contributes to enhanced lipid accumulation under rhythmic light conditions, whereas constant darkness may impair temporal coordination of hepatic lipid metabolism.

The effects of light environments on metabolic physiological mechanisms have been widely demonstrated in mammals [35,36], but their regulatory influence on lipid metabolism genes in chicken embryos remains unclear. Previous studies have shown that, compared to a 1L:23D light regime, a 12L:12D light-dark cycle increases the expression of *SREBP-1c* and TG accumulation in quail liver [17]. As a key transcription factor regulating lipid synthesis, *SREBP-1c* directly modulates the expression of fatty acid synthesis genes such as *ACC*, *FAS*, *SCD1*, and *ELOVL6* [37,38]. Our results showed that, compared to the DD group, the LD group exhibited significantly higher expression levels of *SREBP-1c* and its downstream target genes (*ACC*, *FAS*, *SCD1*, and *ELOVL6*) at specific embryonic stages, further suggesting that rhythmic light cycles may promote lipid synthesis via the *SREBP-1c* pathway. Studies have shown that the circadian clock genes *CLOCK* and *BMAL1* form a complex that activates the transcription of *PPARα*, a key transcription factor involved in fatty acid oxidation, thereby promoting *CPT1* expression and reducing fat deposition [39]. Additionally, mutations in the *CLOCK* gene in intestinal epithelial cells lead to increased expression of the lipolysis-related gene *MTTP* [40]. Consistent with these findings, our study revealed that at specific embryonic stages, the expression levels of *PPARα*, *MTTP*, and *CPT1* were significantly lower in the LD group than in the DD group. This suggests that 12L:12D rhythmic light stimulation may regulate circadian clock gene expression, thereby downregulating the lipid catabolism-related genes *PPARα*, *MTTP*, and *CPT1*, reducing lipid degradation, and promoting hepatic lipid accumulation.

## CONCLUSION

In conclusion, this study elucidates the dynamic effects of rhythmic light exposure during incubation on hepatic development and lipid metabolism in chicken embryos and newly hatched chicks. Our findings suggest that modifying the light cycle, particularly through rhythmic lighting, can effectively enhance embryonic hepatic lipid reserves. This provides a theoretical basis for optimizing incubation protocols and deepens our understanding of the link between circadian rhythms and metabolic regulation in oviparous animals. However, to maximize the benefits of light exposure, further studies are needed to explore the effects of different light cycles, wavelengths, and intensities in order to develop scientifically sound lighting management strategies for poultry production.

## Figures and Tables

**Table 1:** List of primers for lipid metabolism-related genes

Gene	Accession number	Primer sequences (5′ to 3′)	Product size (bp)
*β-actin*	L08165	F: ATTGTCCACCGCAAATGCTTC R: AAATAAAGCCATGCCAATCTCGTC	113
*ACC*	J03541	F: GCTTCCCATTTGCCGTCCTA R: GCCATTCTCACCACCTGATTACTG	185
*FAS*	J03860	F: TTTGGTGGTTCGAGGTGGTA R: CAAAGGTTGTATTTCGGGAGC	215
*CPT1*	AY675193	F: TAGAGGGCGTGGACCAATAA R: TGGGATGCGGGAGGTATT	229
*PPARα*	AF163809	F: TTTAACGGAGTTCCAATCGC R: AACCCTTACAACCTTCACAAGC	224
*SCD1*	NM204890.1	F: GTTTCCACAACTACCACCATACATT R: CCATCTCCAGTCCGCATTTT	175
*SREBP-1c*	XM015294109	F: GCCCTCTGTGCCTTTGTCTTC R: ACTCAGCCATGATGCTTCTTC	130
*ELOVL6*	NM001031539	F: GGTGGTCGGCACCTAATGAA R: TCTGGTCACACACTGACTGC	169
*ChREBP*	EU152408	F: ATTGACCCGACCCTGACG R: CATACTGGATGTACCACGCTCT	160
*MTTP*	NM001109784	F: GCAGATGGACAGAGTTGGCT R: ACACCAAAAGTGCAAGGTGC	224

β-actin, beta-actin; ACC, acetyl CoA carboxylase; FAS, fatty acid synthase; CPT1, carnitine palmitoyltransferase 1; PPARα, peroxisome proliferator-activated receptor α; SCD1, stearoyl-CoA desaturase 1; SREBP-1c, sterol regulatory element binding proteins 1c; ELOVL6, elongase of very long chain fatty acids 6; ChREBP, carbohydrate responsive element binding protein; MTTP, microsomal triglyceride transfer protein.
